# Supplementary material for: Food Insecurity and Socioeconomic Disadvantage in Australia
Source: Int J Environ Res Public Health. 2020 Jan 15;17(2):559. doi: 10.3390/ijerph17020559 (PMC7014009; doi:10.3390/ijerph17020559)
Supplement: Supplementary file 1 [file ijerph-17-00559-s001.pdf]

**Table S1.** Pearson Chi-Square Tests of differences in categorical food security among adults by demographic characteristics.

| | | High food security among adults | Marginal food security among adults | Low food security among adults | Very low food security among adults | Pearson Chi-Square |
|---|---|---|---|---|---|---|
| **Sex** | Male *n* (%) | 13 (10.7) | 5 (4.1) | 20 (16.5) | 83 (68.6) | $\chi^2$ (3, N = 398[1]) = 6.462, $p > 0.05$ |
| | Female *n* (%) | 28 (10.1) | 31 (11.2) | 55 (19.9) | 163 (58.8) | |
| Aboriginal or Torres Strait Islander | Yes *n* (%) | 11 (8.3) | 11 (8.3) | 29 (21.8) | 82 (61.7) | $\chi^2$ (3, N = 400) = 1.875, $p > 0.05$ |
| | No *n* (%) | 30 (11.2) | 25 (9.4) | 46 (17.2) | 166 (62.3) | |
| Marital status | Married or defacto *n* (%) | 3 (5.7) | 7 (13.5) | 14 (26.9) | 28 (53.8) | $\chi^2$ (3, N = 400) = 5.263, $p > 0.05$ |
| | Single *n* (%) | 38 (10.9) | 29 (8.3) | 61 (17.5) | 220 (63.2) | |
| Household composition | Single adult *n* (%) | 13 (12.0) | 6 (5.5) | 20 (18.5) | 69 (63.9) | $\chi^2$ (9, N = 388[2]) = 10.336, $p > 0.05$ |
| | Two or more adults, no children *n* (%) | 4 (5.3) | 5 (6.6) | 12 (15.8) | 55 (72.4) | |
| | Single adult, one or more children *n* (%) | 10 (9.5) | 13 (12.4) | 19 (18.1) | 63 (60.0) | |
| | Two or more adults, one or more children *n* (%) | 12 (12.1) | 11 (11.1) | 23 (23.2) | 53 (53.5) | |
| Educational attainment | Completed high school *n* (%) | 23 (10.1) | 21 (9.3) | 53 (23.3) | 130 (57.3) | **$\chi^2$ (3, N = 400) = 7.857, $p < 0.05$, V=.140** |
| | Did not complete high school *n* (%) | 18 (10.4) | 15 (8.7) | 22 (12.7) | 118 (68.2) | |
| Labor force status | Employed *n* (%) | 7 (13.5) | 5 (9.6) | 16 (30.8) | 24 (46.2) | $\chi^2$ (6, N = 400) = 11.267, $p > 0.05$ |
| | Unemployed *n* (%) | 4 (5.5) | 4 (5.5) | 12 (16.7) | 52 (72.2) | |
| | Not in labor force *n* (%) | 30 (10.9) | 27 (9.8) | 47 (17.0) | 172 (62.3) | |
| Welfare dependent | Yes *n* (%) | 32 (10.6) | 28 (9.3) | 50 (16.6) | 191 (63.5) | $\chi^2$ (3, N = 400) = 3.678, $p > 0.05$ |
| | No *n* (%) | 9 (9.0) | 8 (8.1) | 25 (25.3) | 57 (57.6) | |

[1] Excludes 'other' sex category, [2] Excludes participants with missing data for this variable. Significant results are indicated with bold text.

**Table S2.** Pearson Chi-Square Tests of differences in binary food security among adults by demographic characteristics.

| | | Food security among adults | Food insecurity among adults | Pearson Chi-Square |
|---|---|---|---|---|
| **Sex *n* (%)** | Male | 18 (14.9) | 103 (85.1) | $\chi^2$ (1, N = 398[1]) = 2.227, $p > 0.05$ |
| | Female | 59 (21.3) | 218 (78.7) | |
| Aboriginal or Torres Strait Islander *n* (%) | Yes | 22 (16.5) | 111 (83.5) | $\chi^2$ (1, N = 400) = .940, $p > 0.05$ |
| | No | 55 (20.6) | 212 (79.4) | |
| Marital status *n* (%) | Married or defacto | 10 (19.2) | 42 (80.8) | |

| | | | | |
|---|---|---|---|---|
| | Single | 67 (19.3) | 281 (80.8) | $\chi^2$ (1, N = 400) = .000, p > 0.05 |
| Household composition *n* (%) | Single adult | 19 (17.6) | 89 (82.4) | $\chi^2$ (1, N = 388[2]) = 4.383, p > 0.05 |
| | Two or more adults, no children | 9 (11.8) | 67 (88.2) | |
| | Single adult, one or more children | 23 (21.9) | 82 (88.1) | |
| | Two or more adults, one or more children | 23 (23.2) | 76 (76.8) | |
| Educational attainment *n* (%) | Completed high school | 44 (19.4) | 183 (80.6) | $\chi^2$ (1, N = 400) = .006, p > 0.05 |
| | Did not complete high school | 33 (19.1) | 140 (80.9) | |
| Labor force status *n* (%) | Employed | 12 (23.1) | 40 (76.9) | $\chi^2$ (2, N = 400) = 3.907, p > 0.05 |
| | Unemployed | 8 (11.1) | 64 (88.9) | |
| | Not in labor force | 57 (20.7) | 219 (79.3) | |
| Welfare dependent *n* (%) | Yes | 60 (19.9) | 241 (80.1) | $\chi^2$ (1, N = 400) = .366, p > 0.05 |
| | No | 17 (17.2) | 82 (82.8) | |

[1] Excludes 'other' sex category, [2] Excludes participants with missing data for this variable. Significant results are indicated with bold text

**Table S3.** Pearson Chi-Square Tests of differences in categorical food security among children by demographic characteristics.

| | | High or marginal food security among children | Low food security among children | Very low food security among children | Pearson Chi-Square |
|---|---|---|---|---|---|
| **Sex** | Male *n* (%) | 13 (44.8) | 15 (51.7) | 1 (3.4) | $\chi^2$ (2, N = 216) = 1.994, p > 0.05 |
| | Female *n* (%) | 77 (41.2) | 87 (46.5) | 23 (12.3) | |
| Aboriginal or Torres Strait Islander | Yes *n* (%) | 34 (45.3) | 32 (42.7) | 9 (12.0) | $\chi^2$ (2, N = 216) = .957, p > 0.05 |
| | No *n* (%) | 56 (39.7) | 70 (49.6) | 15 (10.6) | |
| Marital status | Married or defacto *n* (%) | 17 (48.6) | 16 (45.7) | 2 (5.7) | $\chi^2$ (2, N = 216) = 1.593, p > 0.05 |
| | Single *n* (%) | 73 (40.3) | 86 (47.5) | 22 (12.2) | |
| Household composition | Single adult *n* (%) | 2 (66.6) | 1 (33.3) | 0 (0.0) | $\chi^2$ (6, N = 215[1]) = 1.593, p > 0.05 |
| | Two or more adults, no children *n* (%) | 2 (20.0) | 4 (40.0) | 4 (40.0) | |
| | Single adult, one or more children *n* (%) | 45 (43.3) | 49 (47.1) | 10 (9.6) | |
| | Two or more adults, one or more children *n* (%) | 41 (41.8) | 47 (48.0) | 10 (10.2) | |
| Educational attainment | Completed high school *n* (%) | 48 (36.9) | 67 (51.5) | 15 (11.5) | $\chi^2$ (2, N = 216) = 3.105, p > 0.05 |
| | Did not complete high school *n* (%) | 42 (48.8) | 35 (40.7) | 9 (10.5) | |
| Labor force status | Employed *n* (%) | 13 (37.1) | 19 (54.3) | 3 (8.6) | $\chi^2$ (4, N = 216) = 3.802, p > 0.05 |
| | Unemployed *n* (%) | 4 (23.5) | 10 (58.8) | 3 (17.6) | |
| | Not in labor force *n* (%) | 73 (44.5) | 73 (44.5) | 18 (11.0) | |
| | Yes *n* (%) | 74 (45.7) | 69 (42.6) | 19 (11.7) | |

| | | | | | |
|---|---|---|---|---|---|
| Welfare dependent | No *n* (%) | 16 (29.6) | 33 (61.1) | 5 (9.3) | $\chi^2$ (2, N = 216) = 5.667, p > 0.05 |

[1] Excludes participants with missing data for this variable.

**Table S4.** Pearson Chi-Square Tests of differences in binary food security among children by demographic characteristics.

| | | Food security among children | Food insecurity among children | Pearson Chi-Square |
|---|---|---|---|---|
| **Sex *n* (%)** | Male | 13 (44.8) | 16 (55.2) | $\chi^2$ (1, N = 216) = .138, p > 0.05 |
| | Female | 77 (41.2) | 110 (58.8) | |
| Aboriginal or Torres Strait Islander *n* (%) | Yes | 34 (45.3) | 41 (54.7) | $\chi^2$ (1, N = 216) = .636, p > 0.05 |
| | No | 56 (39.7) | 85 (60.3) | |
| Marital status *n* (%) | Married or defacto | 17 (48.6) | 18 (51.4) | $\chi^2$ (2, N = 216) = .819, p > 0.05 |
| | Single | 73 (40.3) | 108 (59.7) | |
| Household composition *n* (%) | Single adult | 2 (66.6) | 1 (33.3) | $\chi^2$ (3, N = 215[1]) = 2.807, p > 0.05 |
| | Two or more adults, no children | 2 (20.0) | 8 (80.0) | |
| | Single adult, one or more children | 45 (43.3) | 59 (56.7) | |
| | Two or more adults, one or more children | 41 (41.8) | 57 (58.2) | |
| Educational attainment *n* (%) | Completed high school | 48 (36.9) | 82 (63.1) | $\chi^2$ (1, N = 216) = 3.023, p > 0.05 |
| | Did not complete high school | 42 (48.8) | 44 (51.1) | |
| Labor force status *n* (%) | Employed | 13 (23.6) | 22 (76.4) | $\chi^2$ (2, N = 216) = 3.142, p > 0.05 |
| | Unemployed | 4 (23.5) | 13 (76.5) | |
| | Not in labor force | 73 (44.5) | 91 (55.5) | |
| Welfare dependent *n* (%) | Yes | 74 (45.7) | 88 (54.3) | **$\chi^2$ (1, N = 216) = 4.292, p < 0.05 V=.141** |
| | No | 16 (29.6) | 38 (70.4) | |

[1] Excludes participants with missing data for this variable. Significant results are indicated with bold text

**Table S5.** Pearson Chi-Square Tests of differences in categorical household food security by demographic characteristics.

| | | High household food security | Marginal household food security | Low household food security | Very low household food security | Pearson Chi-Square |
|---|---|---|---|---|---|---|
| **Sex** | Male *n* (%) | 13 (10.7) | 5 (4.1) | 21 (17.4) | 82 (67.8) | **$\chi^2$ (3, N = 398[1]) = 9.838, p < 0.05, V = .157** |
| | Female *n* (%) | 22 (7.9) | 29 (10.5) | 73 (26.4) | 153 (55.2) | |
| Aboriginal or Torres Strait Islander | Yes *n* (%) | 10 (7.5) | 10 (7.5) | 33 (24.8) | 80 (60.2) | $\chi^2$ (3, N = 400) = .744, p > 0.05 |
| | No *n* (%) | 25 (9.3) | 24 (9.0) | 61 (22.8) | 157 (58.8) | |
| Marital status | Married or defacto *n* (%) | 2 (3.8) | 6 (11.5) | 22 (42.3) | 22 (42.3) | **$\chi^2$ (3, N = 400) = 14.184, p < 0.05, V = .188** |
| | Single *n* (%) | 33 (9.5) | 28 (8.0) | 72 (20.7) | 215 (61.8) | |
| Household composition | Single adult *n* (%) | 13 (12.0) | 6 (5.5) | 20 (18.5) | 69 (63.9) | $\chi^2$ (3, N = 388[2]) = |

| | | | | | | |
|---|---|---|---|---|---|---|
| | Two or more adults, no children *n* (%) | 4 (5.3) | 5 (6.6) | 12 (15.8) | 55 (72.4) | 10.336, p > 0.05 |
| | Single adult, one or more children *n* (%) | 10 (9.5) | 13 (12.4) | 19 (18.1) | 63 (60.0) | |
| | Two or more adults, one or more children *n* (%) | 12 (12.1) | 11 (11.1) | 23 (23.2) | 53 (53.5) | |
| Educational attainment | Completed high school *n* (%) | 20 (8.8) | 17 (7.5) | 63 (27.8) | 127 (55.9) | $\chi^2$ (3, N = 400) = 5.640 p > 0.05 |
| | Did not complete high school *n* (%) | 15 (8.7) | 17 (9.8) | 31 (17.9) | 110 (63.6) | |
| Labor force status | Employed *n* (%) | 6 (11.5) | 4 (7.7) | 19 (36.3) | 23 (44.2) | $\chi^2$ (6, N = 400) = 12.331 p > 0.05 |
| | Unemployed *n* (%) | 4 (5.5) | 3 (4.2) | 13 (18.1) | 52 (72.2) | |
| | Not in labor force *n* (%) | 25 (9.1) | 27 (9.8) | 62 (22.5) | 162 (58.7) | |
| Welfare dependent | Yes *n* (%) | 27 (9.0) | 27 (9.0) | 66 (21.9) | 181 (60.1) | $\chi^2$ (3, N = 400) = 1.824, p > 0.05 |
| | No *n* (%) | 8 (8.1) | 7 (7.1) | 28 (28.3) | 56 (56.6) | |

[1] Excludes 'other' sex category, [2] Excludes participants with missing data for this variable. Significant results are indicated with bold text

**Table S6.** Pearson Chi-Square Tests of differences in binary household food security by demographic characteristics.

| | | Household food security | Household food insecurity | Person Chi-Square |
|---|---|---|---|---|
| **Sex *n* (%)** | Male | 18 (14.9) | 103 (85.1) | $\chi^2$ (1, N = 398[1]) = .735, p > 0.05 |
| | Female | 51 (18.4) | 226 (81.6) | |
| Aboriginal or Torres Strait Islander *n* (%) | Yes | 20 (15.0) | 113 (85.0) | $\chi^2$ (1, N = 400) = .683, p > 0.05 |
| | No | 49 (18.4) | 218 (81.6) | |
| Marital status *n* (%) | Married or defacto | 8 (15.4) | 44 (84.6) | $\chi^2$ (1, N = 400) = .146, p > 0.05 |
| | Single | 61 (17.5) | 287 (82.5) | |
| Household composition *n* (%) | Single adult | 19 (17.6) | 89 (82.4) | $\chi^2$ (3, N = 388[2]) = 4.383, p > 0.05 |
| | Two or more adults, no children | 9 (11.8) | 67 (88.2) | |
| | Single adult, one or more children | 23 (21.9) | 82 (78.1) | |
| | Two or more adults, one or more children | 23 (23.2) | 76 (76.8) | |
| Educational attainment *n* (%) | Completed high school | 37 (16.3) | 190 (83.7) | $\chi^2$ (1, N = 400) = .332, p > 0.05 |
| | Did not complete high school | 32 (18.5) | 141 (81.5) | |
| Labor force status *n* (%) | Employed | 10 (19.2) | 42 (80.8) | $\chi^2$ (2, N = 400) = 3.490, p > 0.05 |
| | Unemployed | 7 (9.7) | 65 (90.3) | |
| | Not in labor force | 52 (18.8) | 224 (81.2) | |
| Welfare dependent *n* (%) | Yes | 54 (17.9) | 247 (82.1) | $\chi^2$ (1, N = 400) = .406, p > 0.05 |
| | No | 15 (15.2) | 84 (84.8) | |

[1] Excludes other' sex category,[2] Excludes participants with missing data for this variable.
